# I-*κ*B Kinase-*ε* Deficiency Attenuates the Development of Angiotensin II-Induced Myocardial Hypertrophy in Mice

**DOI:** 10.1155/2021/6429197

**Published:** 2021-02-08

**Authors:** Yide Cao, Liangpeng Li, Yafeng Liu, Ganyi Chen, Zhonghao Tao, Rui Wang, Wen Chen

**Affiliations:** Department of Thoracic and Cardiovascular Surgery, Nanjing First Hospital, Nanjing Medical University, Changle Road 68, Nanjing, Jiangsu, China

## Abstract

I-*κ*B kinase-*ε* (IKK*ε*) is a member of the IKK complex and a proinflammatory regulator that is active in many diseases. Angiotensin II (Ang II) is a vasoconstricting peptide hormone, and Ang II-induced myocardial hypertrophy is a common cardiovascular disease that can result in heart failure. In this study, we sought to determine the role of IKK*ε* in the development of Ang II-induced myocardial hypertrophy in mice. Wild-type (WT) and IKK*ε*-knockout (IKK*ε*-KO) mice were generated and infused with saline or Ang II for 8 weeks. We found that WT mouse hearts have increased IKK*ε* expression after 8 weeks of Ang II infusion. Our results further indicated that IKK*ε*-KO mice have attenuated myocardial hypertrophy and alleviated heart failure compared with WT mice. Additionally, Ang II-induced expression of proinflammatory and collagen factors was much lower in the IKK*ε*-KO mice than in the WT mice. Apoptosis and pyroptosis were also ameliorated in IKK*ε*-KO mice. Mechanistically, IKK*ε* bound to extracellular signal-regulated kinase (ERK) and the mitogen-activated protein kinase p38, resulting in MAPK/ERK kinase (MEK) phosphorylation, and IKK*ε* deficiency inhibited the phosphorylation of MEK-ERK1/2 and p38 in mouse heart tissues after 8 weeks of Ang II infusion. The findings of our study reveal that IKK*ε* plays an important role in the development of Ang II-induced myocardial hypertrophy and may represent a potential therapeutic target for the management of myocardial hypertrophy.

## 1. Introduction

Heart failure is a growing public health problem and a leading cause of morbidity and mortality in modern society. Pathological cardiac hypertrophy induced by aging and neurohumoral activation (e.g., angiotensin II (Ang II)) is an independent risk factor for heart failure [1, 2]. In fact, clinical and epidemiological studies have identified that cardiac hypertrophy is an important independent risk factor for the development of heart failure and malignant arrhythmia [3]. Initially, cardiac hypertrophy arises as an adaptive response to various common disease stimuli, such as longstanding hypertension, valvular insufficiency, valvular stenosis, myocardial infarction, and coronary artery disease. However, sustained or excessive cardiac hypertrophy can progress to contractile dysfunction and cardiac decompensation, eventually leading to heart failure, arrhythmia, and sudden death [4]. Recently, the multiple signaling mechanisms that control cardiomyocyte growth have been studied extensively, but the molecular mechanisms that mediate the development of cardiac hypertrophy and the transition to heart failure remain incompletely understood.

I-*κ*B kinases (IKKs) have been recognized as regulators of the NF-*κ*B pathway. The IKK complex consists of IKK*α*, IKK*β*, IKK*γ*, and IKK*ε*. IKK*β* and IKK*α* are key regulators of the classical and alternative NF-*κ*B pathways, respectively. IKK*γ*, which is also named NF-*κ*B essential modulator (NEMO), has been confirmed as a regulatory subunit of the NF-*κ*B pathway. IKK*ε* functions in immune and inflammatory responses, tumor growth, and autophagy [5–10]. To date, little is known about the role of IKK*ε* in cardiovascular diseases. Our previous studies have shown that IKK*ε* plays significant roles in both atherosclerotic lesions and aortic stenosis [11, 12]. However, the role of IKK*ε* in the development of myocardial hypertrophy remains unknown.

In this study, we focused on the function of IKK*ε* in Ang II-induced murine myocardial hypertrophy. We found that IKK*ε*-KO attenuated the development of murine myocardial hypertrophy and heart failure induced by Ang II. Additionally, IKK*ε*-KO reduced inflammatory reactions, collagen deposition, apoptosis, and pyroptosis in murine heart tissue. Analysis of the signaling pathway demonstrated that IKK*ε*-KO inhibited the phosphorylation of the MAPK pathway proteins MEK1/2-ERK1/2 and p38 in the heart tissue infused with Ang II for 8 weeks. Taken together, our results indicate that IKK*ε* promotes the development of Ang II-induced murine myocardial hypertrophy and that IKK*ε* is a potential therapeutic target for the treatment of myocardial hypertrophy.

## 2. Results

### 2.1. IKK*ε* Was Evaluated in Mice with Myocardial Hypertrophy

**Figure 1:**
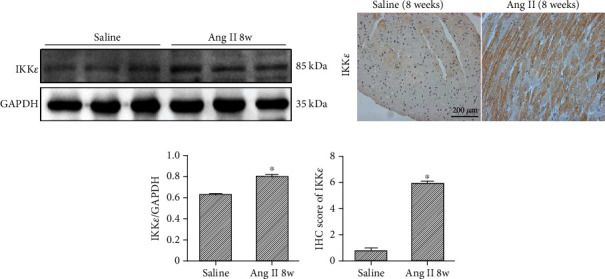
The expression of IKK*ε* was increased in WT mouse hearts after infusion of Ang II for 8 weeks. (a) Representative western blot showing the expression of IKK*ε* in the heart tissue at the 8^th^ week of Ang II infusion (*n* = 4 mice per experimental group). (b) Representative images of IHC staining showing IKK*ε* in WT mouse hearts after 8 weeks of Ang II stimulation. (*n* = 4 mice per experimental group, 400x; ^∗^*P* < 0.05 vs. saline). (c, d) Quantitative western blot and IHC analyses of IKK*ε* (^∗^*P* < 0.05 vs. saline).

Initially, we established a myocardial hypertrophy model in mice by infusion of angiotensin II. We found an obvious increase in IKK*ε* in WT mouse heart tissues after 8 weeks of continuous Ang II infusion. Western blotting and IHC staining showed increased expression of IKK*ε* (Figure 1).

### 2.2. IKK*ε* Knockout Attenuated the Development of Ang II-Induced Myocardial Hypertrophy

**Figure 2:**
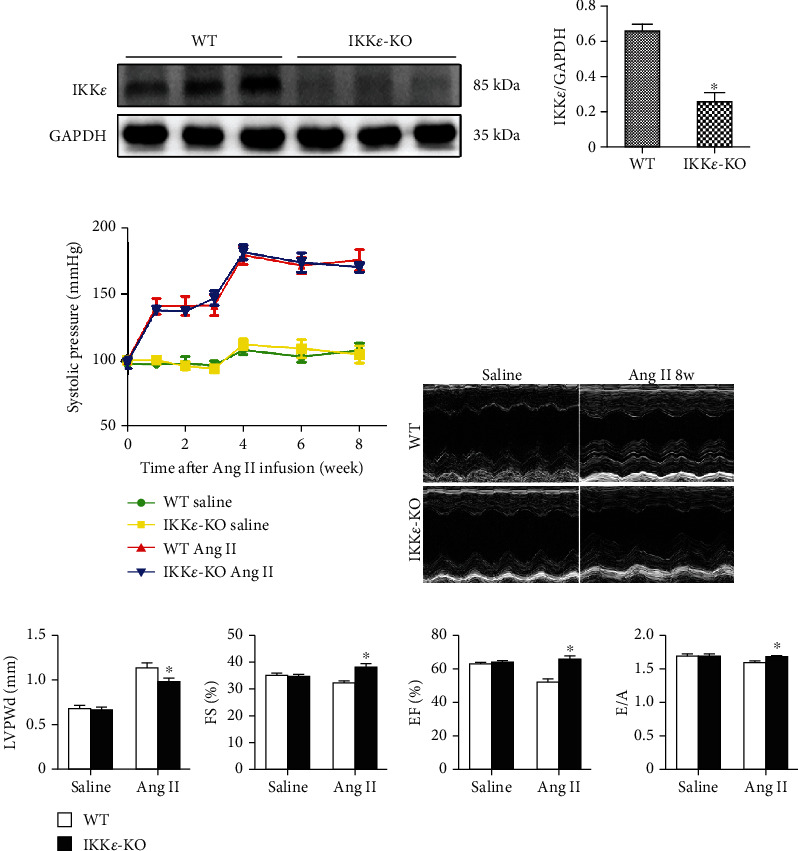
IKK*ε* deficiency attenuated the cardiac dysfunction after Ang II stimulation. (a) Representative images and quantitative analyses of western blot showing the expression of IKK*ε* in the heart tissue of WT and IKK*ε*-KO mice (^∗^*P* < 0.05 vs. WT). (b) Changes in blood pressure after Ang II infusion. (c) Representative images showing the echocardiography of WT and IKK*ε*-KO mice that were infused with saline or Ang II for 8 weeks. (d) Analysis of echocardiography parameters (LVPWd, EF, FS, and E/A) of WT and IKK*ε*-KO mice that were infused with saline or Ang II for 8 weeks (*n* = 6 mice per experimental group; ^∗^*P* < 0.05 vs. saline or WT Ang II).

**Figure 3:**
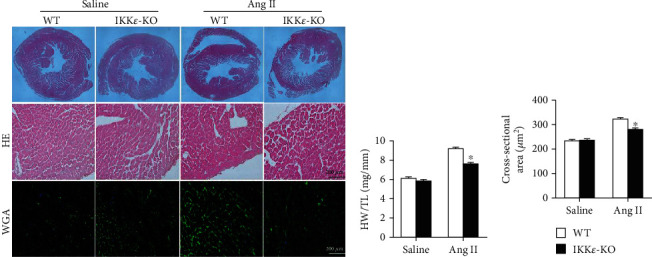
IKK*ε* deficiency alleviated the development of Ang II-induced myocardial hypertrophy. (a) Representative images showing panoramagrams, HE staining and WGA staining of WT and IKK*ε*-KO mice (*n* = 4 mice per experimental group). (b, c) Analyses of heart weight/tibial length (HW/TL) and cross-sectional area of WT and IKK*ε*-KO mice under Ang II stimulation (*n* = 10 mice per experimental group in HW/TL; *n* = 4 mice per experimental group in a cross-sectional area, 400x in HE and WGA; ^∗^*P* < 0.05 vs. saline or WT Ang II).

First, we detected the protein level of IKK*ε* to confirm gene knockout in the mice. Western blotting of proteins from WT and IKK*ε*-KO mouse heart tissues showed the successful knockout of IKK*ε* in IKK*ε*-KO mice (Figure 2(a)). To examine the role of IKK*ε* during hypertrophy in vivo, we infused WT and IKK*ε* knockout mice with Ang II or saline through subcutaneous implantation of miniosmotic pumps containing Ang II (1 *μ*g/kg/min) in saline or saline alone. It is important to note that at baseline, the IKK*ε* knockout mice were viable and fertile and had no pathological alterations in their heart morphology or contractile function. Initially, we tested the change in pressure in mice after Ang II infusion. We found an obvious increase in systolic pressure from the 1^st^ week to the 4^th^ week of infusion, and the pressure did not significantly increase from the 4^th^ week to the 8^th^ week (Figure 2(b)). The echocardiographic analysis of cardiac function showed a significant decrease in the EF, FS, and E/A, as well as an increase in the left ventricular posterior wall diastole (LVPWd) in WT mice after 8 weeks of Ang II infusion, which indicated the attenuation of myocardial hypertrophy and deterioration of LV function in IKK*ε*-KO mice compared with those of WT mice (Figures 2(c) and 2(d)). Additionally, after 8 weeks of Ang II infusion, WT mouse hearts exhibited significant hypertrophic deterioration, as indicated by HE staining showing increased gross heart size (Figure 3(a)), as well as increased cardiomyocyte cross-sectional area (by wheat germ agglutinin staining) and increased ratios of heart weight (HW)/tibial length (TL) (Figures 3(b) and 3(c)), compared with those of Ang II-induced IKK*ε*-deficient hearts.

### 2.3. IKK*ε* Knockout Alleviated Heart Failure, Inflammation, and Collagen Deposition after Ang II Induction

**Figure 4:**
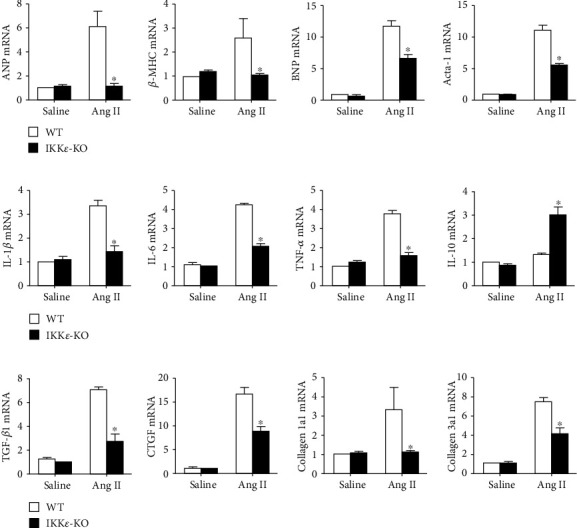
IKK*ε* knockout decreased the inflammatory reaction and fibrosis in Ang II-induced myocardial hypertrophy. Relative mRNA levels of heart failure markers (a) (atriopeptin, brain natriuretic peptide, Acta-1, and *β*-MHC), inflammatory factors (b) (IL-6, TNF-*α*, IL-1*β*, and IL-10), and fibrosis-related factors (c) (TGF-*β*1, CTGF, Collagen 1a1, and Collagen 3a1) in the heart tissues of WT or IKK*ε*-KO mice after Ang II infusion (*n* = 4 mice per experimental group; ^∗^*P* < 0.05 vs. saline or WT Ang II).

**Figure 5:**
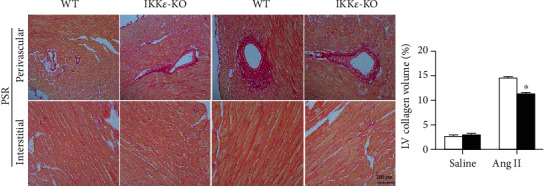
IKK*ε* knockout reduced collagen deposition in Ang II-induced myocardial hypertrophy. Representative images showing picrosirius red staining in perivascular and interstitial areas of the heart tissue from WT and IKK*ε*-KO mice after Ang II stimulation and the related quantitative analyses of left ventricular collagen volume (*n* = 4 mice per experimental group, 400x; ^∗^*P* < 0.05 vs. saline or WT Ang II).

Subsequently, we performed further research on the role of IKK*ε* in pathophysiology. PCR analysis showed significant decreases in heart failure markers (atriopeptin (ANP), brain natriuretic peptide (BNP), Acta-1, and *β*-MHC), proinflammatory factors (IL-6, TNF-*α*, and IL-1*β*), and fibrosis markers (connective tissue growth factor (CTGF), Collagen 1a1, and Collagen 3a1) in IKK*ε*-KO mice compared with those of WT mice (Figures 4(a)–4(c)). Picrosirius red staining revealed that the collagen volume in both the interstitial and perivascular spaces of the left ventricle was significantly reduced in IKK*ε*-KO mice (Figure 5). The results indicated that IKK*ε* alleviated heart failure, collagen deposition, and inflammatory reactions after 8 weeks of Ang II infusion.

### 2.4. The Lack of IKK*ε* Ameliorated Apoptosis and Pyroptosis in Myocardial Tissue after Ang II Infusion

**Figure 6:**
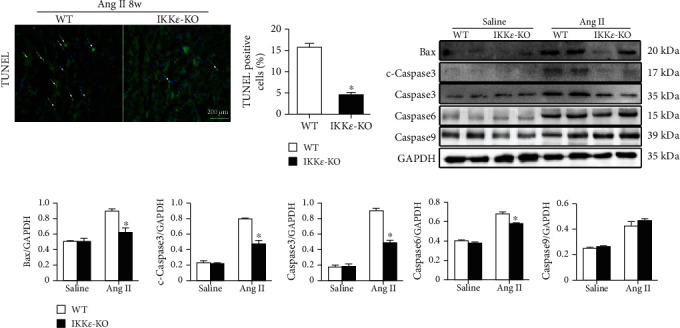
The lack of IKK*ε* reduced apoptosis in myocardial tissue after Ang II infusion. (a) Representative images and TUNEL assay of WT and IKK*ε*-KO mice after 8 weeks of Ang II infusion (*n* = 4 mice per experimental group, 400x; ^∗^*P* < 0.05 vs. WT). (b, c) Representative western blots and quantitative analyses of apoptosis-related proteins in the heart tissue of WT and IKK*ε*-KO mice that were infused with Ang II for 8 weeks (*n* = 4 mice per experimental group, ^∗^*P* < 0.05 vs. saline or WT Ang II).

**Figure 7:**
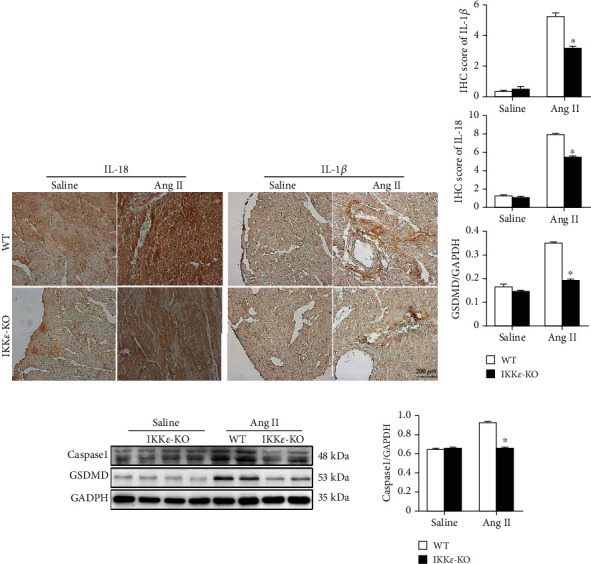
The lack of IKK*ε* reduced pyroptosis in myocardial tissue after Ang II infusion. (a, b) Representative IHC images and analyses of IL-1*β* and IL-18 in WT and IKK*ε*-KO mouse heart tissue after 8 weeks of Ang II infusion (*n* = 4 mice per experimental group, 400x, ^∗^*P* < 0.05 vs. saline or WT Ang II). (c) Representative western blots and quantitative analyses of pyroptosis-related proteins in the heart tissue of WT and IKK*ε*-KO mice that were infused with Ang II for 8 weeks (*n* = 4 mice per experimental group, ^∗^*P* < 0.05 vs. saline or WT Ang II).

After the detection of inflammatory factors, we tested apoptosis- and pyroptosis-related factors in myocardial tissue. The results of TUNEL assays and analysis of apoptosis-related proteins (except Caspase9) revealed that apoptosis in WT mice was more severe than that in IKK*ε*-KO mice (Figures 6(a)–6(c)). Moreover, IHC results showed significantly increased expression of IL-1*β* and IL-18 in WT mice compared to that of IKK*ε*-KO mice (Figures 7(a) and 7(b)). Additionally, western blot results of pyroptosis-related proteins (caspase1 and GSDMD) showed the same trend (Figure 7(c)). These results demonstrated that the lack of IKK*ε* ameliorated apoptosis and pyroptosis in Ang II-induced myocardial hypertrophy.

### 2.5. IKK*ε* Deficiency Inhibits the Ang II-Mediated MEK1/2-ERK1/2 and P38 Signaling Pathways

**Figure 8:**
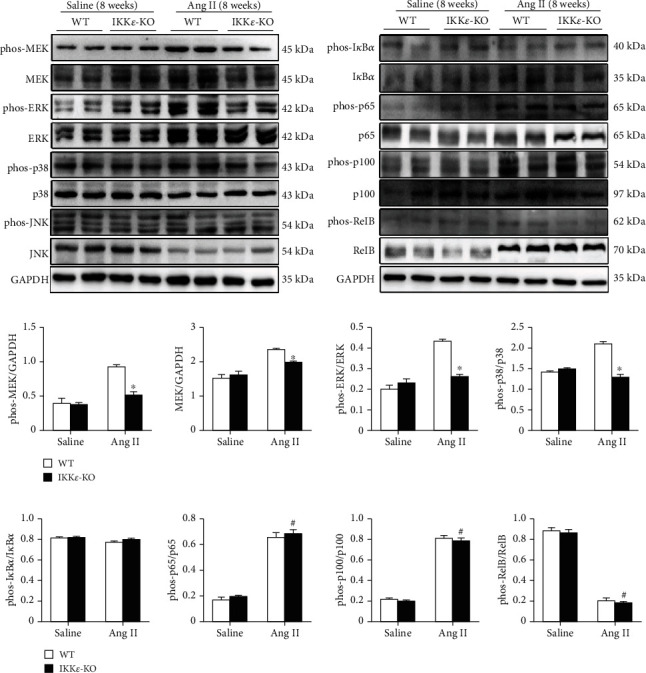
IKK*ε* deficiency inhibits the Ang II-mediated MEK1/2-ERK1/2 and P38 signaling pathways. (a, b) Representative western blots showing the phosphorylation and total protein levels of the MAPK pathway proteins MEK1/2, ERK1/2, JNK, and p38 and the NF-*κ*B pathway proteins I*κ*B*α*, p65, RelB, and p100 in the heart tissue of WT and IKK*ε*-KO mice that were infused with Ang II for 8 weeks (*n* = 6 independent experiments). (c) Relative quantitative analyses of western blot analysis of the MAPK pathway (*n* = 6 mice per experimental group; ^∗^*P* < 0.05 vs. saline or WT Ang II). (d) Relative quantitative analyses of western blot analysis of the NF-*κ*B pathway (*n* = 6 mice per experimental group; ^#^*P* < 0.05 vs. saline).

**Figure 9:**
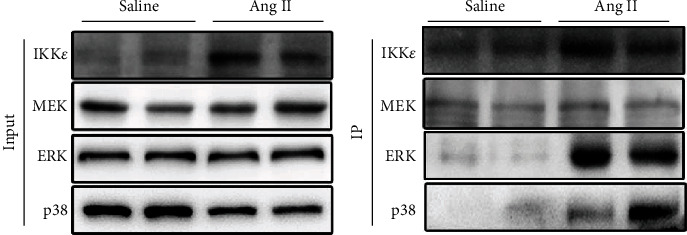
IKK*ε* combined with ERK and p38 in the heart tissue under Ang II stimulation. Coimmunoprecipitation analysis of IKK*ε*, MEK, ERK, and p38 in the heart tissues of WT mice that were stimulated by saline or Ang II for 8 weeks.

Due to the significant differences in inflammatory reactions, we tested two common pathways that are associated with inflammation, the MAPK and NF-*κ*B pathways, in WT and IKK*ε*-KO mouse hearts after infusion with Ang II for 8 weeks. We discovered that there were no significant differences in the NF-*κ*B pathway between WT and IKK*ε*-KO mice that were infused with saline or Ang II (Figure 8(b)). Interestingly, we found obvious differences in the MEK1/2-ERK1/2 and p38 pathways between these two groups after 8 weeks of Ang II infusion. As shown in Figures 8(a) and 8(c), IKK*ε*-KO inhibited the phosphorylation of MEK1/2, ERK1/2, and p38 in the heart after infusion with Ang II for 8 weeks. To further explore the mechanism by which IKK*ε* regulates Ang II-induced myocardial hypertrophy, we performed coimmunoprecipitation of IKK*ε*, MEK, ERK, and p38 in the heart tissues of WT mice that were stimulated with saline or Ang II for 8 weeks. The results in Figure 9 show that IKK*ε* was bound to ERK and p38 of the MAPK pathway in the heart tissue after 8 weeks of Ang II stimulation. In conclusion, IKK*ε* regulated Ang II-induced myocardial hypertrophy mainly through the MAPK pathway. Furthermore, IKK*ε* binding to ERK and p38 and was involved in MEK phosphorylation.

## 3. Discussion

In the present study, we elucidated the role of IKK*ε* in Ang II-induced myocardial hypertrophy using WT and IKK*ε* mice. Our results revealed that knockout of IKK*ε* protected the hearts against maladaptive hypertrophy, dilatation, fibrosis, inflammation, apoptosis, pyroptosis, and pathological cardiac remodeling in response to long-term Ang II infusion. Mechanistically, we demonstrated that IKK*ε* bound to ERK and p38 and inhibited Ang II-induced myocardial hypertrophy by downregulating the MEK1/2-ERK1/2 and p38 signaling pathways. Thus, we provide the first evidence indicating that IKK*ε* plays a critical role in exacerbating Ang II-induced myocardial hypertrophy.

Myocardial hypertrophy is a common cardiac disease, especially in elderly individuals and patients with hypertension [13]. Cardiac failure can easily occur after myocardial hypertrophy is diagnosed. Hypertension is characterized by cardiac hypertrophy and dysfunction [14]. The systolic pressure of the mice was obviously increased after Ang II infusion, especially in the first 4 weeks. However, the increased blood pressure was not significantly different between WT and IKK*ε*-KO mice during the course of the infusion, which meant that the differences in myocardial hypertrophy between the two groups were not related to blood pressure. The differences in myocardial hypertrophy may be due to the role of Ang II in cardiomyocytes. Therefore, we examined whether IKK*ε* inhibition has cardioprotective effects in Ang II-induced myocardial hypertrophy. Our echocardiographic data showed that hypertensive mice exhibited cardiac hypertrophy and impaired cardiac function. However, IKK*ε*-KO mice had a thinner left ventricular wall than that of WT mice. Additionally, IKK*ε* inhibition increased EF, FS, and E/A after 8 weeks of Ang II infusion, which indicated improved cardiac function in IKK*ε*-KO mice compared to that of WT mice with Ang II-induced myocardial hypertrophy.

Cardiomyocyte enlargement is a pathological phenomenon of both physiological and pathological hypertrophies [15]. As shown in Figure 3, HE and WGA staining revealed that the cross-sectional areas of cardiomyocytes were increased after infusion of Ang II for 8 weeks. Moreover, IKK*ε*-KO mice showed a smaller cardiomyocyte area than the WT mice. This demonstrated that IKK*ε*-KO attenuates myocardial hypertrophy in Ang II-induced hypertensive mice. In contrast to our results, Dai et al. [16] demonstrated that IKK*ε*-deficient mice showed significantly enhanced cardiac hypertrophy, cardiac dysfunction, apoptosis, and fibrosis compared with those of WT mice, and these effects were associated with activation of the AKT and NF-*κ*B signaling pathways in response to aortic banding. However, in the present study, we induced myocardial hypertrophy by infusion of Ang II, which resulted in myocardial hypertrophy through different mechanisms. These two different stimulations induce myocardial hypertrophy by different mechanisms. Ang II induces myocardial hypertrophy by pathophysiological and inflammatory mechanisms, while aortic banding induces hypertrophy by a pressure load mechanism. We considered Ang II to be better than aortic banding in inducing murine myocardial hypertrophy by simulating the development of human hypertension and secondary myocardial hypertrophy. Although we researched the same gene in the same disease, the pathomechanisms of the development of myocardial hypertrophy are different in the two researches. Thus, it is reasonable that IKK*ε* plays a different role under different stimulations in the same disease, which also revealed the versatile function of IKK*ε*. Additionally, previous studies in our lab found that the loss of IKK*ε* reduced adverse aortic valve thickening in response to Ang II [12]. IKK*ε* prevented obesity and inflammatory responses in the murine hearts of ApoE(-/-) and ApoE(-/-)/IKK*ε*(-/-) mice that were fed an ND and HFD, respectively [17]. Knockout of IKK*ε* prevented significant atherosclerosis lesions in mouse aortas from both wild-type and ApoE knockout mice that were fed a high-fat diet [11]. These findings demonstrate that IKK*ε* is a detrimental factor in the inflammatory reaction and physiological changes in the heart induced by Ang II.

Myocardial hypertrophy has been associated with apoptosis and pyroptosis in previous studies [18–21]. In our study, we found that IKK*ε* deficiency ameliorated apoptosis and pyroptosis in myocardial hypertrophy. The results of the TUNEL assay and western blot analysis of Bax, cleaved-caspase3, and caspase6 all showed a significant increase in apoptosis in WT mice compared to that of IKK*ε*-KO mice. However, the expression of caspase9 was not different between the two groups. Pyroptosis is a novel kind of cell death that is morphologically and mechanistically distinct from other forms of cell death [22]. In contrast to apoptosis, pyroptosis is a form of necrotic and inflammatory programmed cell death that is induced by inflammatory caspases [23]. Caspase1 dependence is a defining feature of pyroptosis, and cleaved-caspase1 is capable of converting prointerleukin-1*β* (pro-IL-1*β*) and prointerleukin-18 (pro-IL-18) into mature IL-1*β*/IL-18. Recent studies have revealed that gasdermin D (GSDMD) is another critical component of the inflammasome and is cleaved by activated caspase1 [24, 25]. We detected pyroptosis-related proteins and found that the expression of representative factors of pyroptosis (IL-1*β*, IL-18, caspase1, and GSDMD) was higher in WT mice than in IKK*ε*-KO mice. Consequently, IKK*ε* deficiency attenuates apoptosis and pyroptosis in Ang II-induced myocardial hypertrophy.

IKK*ε* is known as a noncanonical IKK and expands the IKK family to four members (IKK*α*, IKK*β*, IKK*γ*, and IKK*ε*) [26, 27]. IKK*ε* is a new member that shares 33% and 31% amino acid identity with the main domains of IKK complex, IKK*α*, and IKK*β*, as well as 67% with TBK1 [28]. Additionally, IKK*ε* is a noncanonical I*κ*B kinase that plays a pivotal role in inflammation, autophagy, cell survival, immunity, proliferation, and transformation [9, 29]. IKK*ε* activity has been linked to the pathology of inflammatory diseases such as rheumatoid arthritis (RA) [30–32]. Consistent with previous studies [30, 33], IKK*ε* knockout inhibits the inflammatory reaction. For instance, IKK*ε*-deficient mice exhibit significantly reduced nociceptive behavior in comparison with that of wild-type mice [34]; mice that are deficient in IKK*ε* were found to be protected from high-fat diet-induced obesity and showed reduced chronic liver inflammation, hepatic steatosis, and insulin resistance [35, 36]. In our study, an increase in anti-inflammatory factors (IL-10) and a decrease in proinflammatory factors (IL6, IL-1*β*, and TNF-*α*) were observed. TGF-*β*1 is considered an anti-inflammatory factor and is highly expressed in WT mice, which can be explained by increased fibrosis and collagen deposition in WT mouse hearts.

IKK*ε* is a member of the IKK complex that regulates the NF-*κ*B pathway. However, IKK*ε* does not always activate the NF-*κ*B pathway. Previous studies have shown that IKK*ε* also activates the AKT and MAPK pathways [7, 37, 38] and even has no relationship with the NF-*κ*B pathway in some conditions [10, 39]. The MAPK and NF-*κ*B pathways are both essential mediators of inflammation. Several studies have demonstrated that the MAPK pathway has a strong relationship with the development of myocardial hypertrophy [40–43]. Furthermore, Bulek et.al revealed that phosphorated ERK was inhibited in IKK*ε* deficiency airway epithelial cells under IL-17A stimulation [33]. Additionally, the last published research of our lab demonstrated that IKK*ε* deficiency blunted the activation of the ERK1/2 pathway in primary mouse aortic VSMC under Ang II stimulation [44]. In our study, we found that knocking out IKK*ε* prevented the activation of MEK-ERK and p38 phosphorylation under Ang II stimulation for 8 weeks. Coimmunoprecipitation assays showed that IKK*ε* bound to ERK and p38 of the MAPK pathway. Although many previous studies have demonstrated that IKK*ε* is strongly linked with the phosphorylation of p65 in the classical NF-*κ*B pathway [17, 34, 45, 46], none of these studies were associated with Ang II stimulation. Therefore, we hypothesized that IKK*ε* has a relationship with the MAPK pathway in the murine heart after infusion with Ang II.

Limited by time, this study only performed in vivo research, which revealed that IKK*ε* is an important regulator in the development of Ang II-induced murine myocardial hypertrophy. We are preparing to conduct more research on the function of IKK*ε* in neonatal rat myocardial cells by knocking down or overexpressing IKK*ε*. Further studies will validate the exact mechanism of IKK*ε* in myocardial cells under Ang II stimulation. In addition, this article was written by Dr. Cao, who also wrote the article “Regulatory Role of IKKɑ in Myocardial Ischemia/Reperfusion Injury by the Determination of M1 versus M2 Polarization of Macrophages,” and these two researches were accomplished by the same laboratory. Thus, it is reasonable that there is significant reuse of wording in the Methods sections in the two articles.

In conclusion, IKK*ε*-KO attenuates Ang II-induced murine myocardial hypertrophy and heart failure and reduces inflammatory reactions, apoptosis, pyroptosis, and collagen deposition by inhibiting the MEK1/2-ERK1/2 and p38 pathways. Furthermore, IKK*ε* binds with ERK and p38 in heart tissues under Ang II stimulation. Consequently, our study provides a novel therapeutic target for the treatment of myocardial hypertrophy.

## 4. Materials and Methods

We followed the most methods of the previous article researched by Cao et al. of our laboratory [47].

### 4.1. Mice

All animal procedures were performed in compliance with the Institute of Laboratory Animal Research Guide for the Care and Use of Laboratory Animals of the National Institutes of Health and were approved by the Institutional Animal Care and Use Committee of Nanjing Medical University (Ethics Committee of Nanjing First Hospital). IKK*ε* knockout mice (B6. Cg-Ikbketm1Tman/J, IKK*ε*-KO) were obtained from Jackson Laboratory (Bar Harbor, ME, USA) and underwent rederivation to achieve pathogen-free status in the Model Animal Research Center of Nanjing University (Nanjing, China). Wild-type mice (C57B6/L) were obtained from the Model Animal Research Center of Nanjing University. All mice in this research were male at the age between 10 and 12 weeks. The mice were infused with Ang II or saline through subcutaneous implantation of miniosmotic pumps (ALZET, DURECT Corp.) containing Ang II (1 *μ*g/kg/min) in saline or saline alone. All mice were housed in specific pathogen-free box cages at room temperature with a 12-hour light/12-hour dark cycle and free access to a normal diet and water. The mice were infused with saline or Ang II for 8 weeks of the experimental period.

### 4.2. Echocardiography Evaluation

Isoflurane (1.5–2%) was used to narcotize mice by inhalation, and echocardiography was then performed using a Vevo 2100 ultrasound with a 30-MHz linear array ultrasound transducer. Left ventricular posterior wall distance (LVPWd), ejection fraction (EF), fraction shortening (FS), and E/A were measured from M-mode tracings with a sweep speed of 50 mm/s at the midpapillary muscle level. End-systole or end-diastole phases corresponded to the smallest or largest LV diameters, respectively. Echocardiographic measurements were taken in M-mode in triplicate from more than 3 separate mice per group.

### 4.3. Histological and Immunohistochemical Staining

Mouse hearts were removed, immediately immersed in 4% neutral phosphate-buffered paraformaldehyde for 12 h, embedded in paraffin. The paraffin blocks were sectioned into slices of 5 *μ*m. The sections were stained with hematoxylin-eosin (HE), Masson's trichrome, picrosirius red (PSR), or wheat germ agglutinin (WGA). The morphological changes, fibrosis, and collagen deposition in the myocardium were observed under a light microscope.

For immunohistochemical staining, paraffin-embedded heart tissues were prepared as 4 *μ*m thick sections and rehydrated in graded alcohol.

Hydrogen peroxide (3%) was used to block endogenous peroxidase activity before incubated in imported goat serum (ZLI-9022, Beijing Zhongshan Biotechnology) to prevent nonspecific binding of antibodies. The sections were then incubated separately for 14 h with antibodies against IKK*ε* (1 : 100, 3416, CST), IL-1*β* (1 : 50, sc7884, Santa Cruz), and IL-18 (1 : 100, ab71495, Abcam). After incubation of primary antibodies, the sections were incubated with goat anti-rabbit or anti-mouse IgG (KIT-5004 and KIT-5001, MXB) for 1 h at 37°C in a wet box. The signal of each antibody came to light by the substrate diaminobenzidine (DAB, ZLI-9018, Beijing Zhongshan Biotechnology). Photomicrographs were taken by a Zeiss Scope.A1 camera. The results of immunohistochemistry were analyzed by Fromowitz semiquantitative analysis score (range: 0 to 7). The average score of each slice was determined by two independent observers.

### 4.4. Total RNA Extraction and Quantitative Real-Time PCR (qRT-PCR)

Total RNA was extracted from heart tissues using TRIzol™ reagent (Invitrogen, 15596-026). Equal quantities of RNA (1 *μ*g) were converted into cDNA with a PrimScript™ RT reagent kit with gDNA Eraser (Takara, RR047A). Quantitative TaqMan PCR was performed with SYBR Premix Ex TaqTM II (Takara, RR082A) tested by an Applied Biosystems 7500 Real Time PCR System. All data were normalized to GAPDH.

### 4.5. Western Blotting

Total protein samples (30 *μ*g) were extracted from left ventricular tissue. The proteins were separated by SDS-PAGE and then transferred to polyvinylidene fluoride (PVDF) membranes (Millipore). Tris-buffered saline (TBS) with Tween diluted at 1 : 1000 (TBST; Promega) was used to wash PVDF membranes for 10 min; then the PVDF membranes were blocked with TBST containing 5% BSA for 1 h. The membranes were incubated with the following primary antibodies in TBST with Tween plus 5% BSA overnight at 4°C: anti-phosphorylated IKK*ε* (1 : 1000, 3416, CST), Bax (1 : 1000, 2772S, CST), Caspase1 (1 : 1000, 2225T, CST), cleaved-Caspase3 (1 : 1000, 9661S, CST), Caspase6 (1 : 1000, 9762T, CST), Caspase9 (1 : 1000, 9508T, CST), GSDMS (1 : 1000, ab219800, Abcam), p65 (1 : 1000, cs3033, CST), anti-p65 (1 : 200; sc8008, Santa Cruz), anti-phosphorylated I*κ*B*α* (1 : 1000, 2859s, CST), anti-I*κ*B*α* (1 : 200; sc371, Santa Cruz), anti-phosphorylated p100/p52 (1 : 500, ab31474, Abcam), anti-p100/p52 (1 : 500, ab109440, Abcam), anti-phosphorylated RelB (1 : 500, ab47366, Abcam), anti-RelB (1 : 1000, ab180127, Abcam), anti-phosphorylated p38 (1 : 1000, 4511s, CST), anti-p38 (1 : 1000, 9212s, CST), anti-phosphorylated JNK (1 : 1000, 4668s, CST), anti-JNK (1 : 1000, 9258s, CST), anti-phosphorylated MEK1/2 (1 : 1000, 9154s, CST), anti-MEK1/2 (1 : 1000, 9122s, CST), anti-phosphorylated ERK1/2 (1 : 1000, 4370s, CST), anti-ERK1/2 (1 : 1000, 4695s, CST), and HRP-conjugated monoclonal mouse anti-glyceraldehyde-3-phosphate dehydrogenase (GAPDH) (1 : 5000, KC-5G5, KangChen). After being incubated overnight, TBST was configurated to wash the PVDF membranes for 30 min (10 min∗3). Subsequently, the PVDF membranes were immersed in goat anti-mouse IgG/HRP (1 : 5000, bs-0296G-HRP, Bioss) or anti-rabbit IgG HRP-linked antibodies (1 : 5000, 7074P2, CST) at room temperature for 1 h. The protein bands were emerged by an Immobilon western chemiluminescent HRP substrate (WBKLS0500, Millipore), and pictures were took with ChemiScope (3300 Mini, Clinx Science Instruments). The gray value of each bolt was then detected by Chemi analysis software.

## 5. Coimmunoprecipitation

For the coimmunoprecipitation assay, the heart tissue proteins of WT mice that were stimulated with saline or Ang II for 8 weeks were precleared for 1 h with the Protein G Magnetic Bead System (LSKMAGG02, Millipore) After that, the proteins were incubated with 3 *μ*g anti-IKK*ε* antibody (1 : 1000, 3416, CST) overnight, followed by the Protein G Magnetic Bead System for an additional hour. An equal amount of protein from each preparation was separated by SDS-PAGE, blotted, and detected with anti-IKK*ε* (1 : 1000, 3416, CST), anti-MEK1/2 (1 : 1000, 9122s, CST), anti-ERK1/2 (1 : 1000, 4695s, CST), and anti-p38 (1 : 1000, 9212s, CST) antibodies. Immunoprecipitates were then washed with 1 ml lysis buffer and boiled with 1x loading buffer. Then, those Immunoprecipitates were separated by SDS-PAGE for immunoblot analysis with anti-IKK*ε* (1 : 1000, 3416, CST), anti-MEK1/2 (1 : 1000, 9122s, CST), anti-ERK1/2 (1 : 1000, 4695s, CST), and anti-p38 (1 : 1000, 9212s, CST) antibodies.

## 6. Statistical Analysis

The data are expressed as the mean ± SE. Differences between groups were evaluated by analysis of variance and Tukey's test after the fact. Student's *t*-test was used for comparison between the two groups. All statistical analyses were performed using SPSS version 17.0 software. *P* < 0.05 means the difference is statistically significant.

## Data Availability

We declare that all data are contained within the manuscript.
